# Physical Activity on Prescription with Counsellor Support: A 4-Year Registry-Based Study in Routine Health Care in Sweden

**DOI:** 10.3390/healthcare6020034

**Published:** 2018-04-16

**Authors:** Pia Andersen, Sara Holmberg, Lena Lendahls, Per Nilsen, Margareta Kristenson

**Affiliations:** 1 Department of Research and Development, Region Kronoberg, 351 88 Växjö, Sweden; sara.holmberg@kronoberg.se (S.H.); lena.lendahls@lnu.se (L.L.); 2 Division of Community Medicine, Department of Medical and Health Sciences, Linköping University, 581 83 Linköping, Sweden; per.nilsen@liu.se (P.N.); margareta.kristenson@liu.se (M.K.); 3 Division of Occupational and Environmental Medicine, Institute of Laboratory Medicine, Lund University, 221 00 Lund, Sweden; 4 Department of Health and Caring Sciences, Faculty of Health and Life Sciences, Linnaeus University, 391 82 Kalmar, Sweden

**Keywords:** physical activity prescription, implementation, counselling, primary care, secondary care

## Abstract

*Background*: Public health gains from physical activity on prescription (PAP) depend on uptake in routine care. We performed an evaluation of the implementation, in a Swedish county council, of counsellors who give personalized support to PAP recipients aimed at facilitating PAP delivery. The aim was to compare characteristics between PAP recipients and the health care population as well as between PAP recipients who used and did not use counsellor support. We also investigated professional belonging and health care setting of health care professionals who prescribed PAP. *Methods:* All patients’ ≥18 years who received PAP during 2009–2012 in primary and secondary care in the County Council of Kronoberg were included (*n* = 4879). Data were retrieved from electronic medical records. Main outcome measures were patient and professional characteristics. *Results:* A third of the PAP recipients had diseases in ≥5 diagnostic groups and more than half had ≥11 office visits the year before receiving PAP. Counsellor support was used by one-third and PAP recipients who used counsellor support had more multiple diagnoses and office visits compared with non-users. Physicians issued 44% of prescriptions and primary care was the predominant setting. The amount of PAP did not change over time, but the proportion of physicians’ prescriptions decreased while the proportion of nurses’ prescriptions increased. *Conclusions:* PAP recipients had high morbidity and were frequent health care attenders, indicating that PAP was predominantly used for secondary or tertiary prevention. PAP rates did not increase as intended after the implementation of counsellor support.

## 1. Background

Insufficient physical activity contributes considerably to premature mortality and is a risk factor for a broad range of non-communicable diseases, including cardiovascular diseases, cancer, diabetes, as well as musculoskeletal and mental health disorders [1,2]. Globally, one in four adults report physical activity below recommended levels, and the proportion is even higher in Sweden: one in three adults [3]. Hence, policies and interventions to achieve increased physical activity levels in the population are of utmost importance for public health [1,2].

The concept of physical activity on prescription (PAP) has been developed for health care services mainly to support patients in primary care who might benefit from increased physical activity [4,5,6,7,8,9]. The Swedish PAP concept (referred to as FaR in Sweden) was introduced in 2001 as a part of the national project “Sweden on the move” and is now used by all Swedish county councils (which are responsible for provision of health care in Sweden). The concept consists of patient-centred counselling, written prescription of individualized physical activity using the FYSS manual (the meaning of “FYSS” is Physical Activity in the Prevention and Treatment of Disease, English version) [10], collaboration with local organizations and follow-up assessments of the recipient’s physical activity [11]. The FYSS manual is an evidence-based handbook for health care professionals and it describes how physical activity can be used in prevention and treatment of several types of diseases and conditions. The PAP concept in Sweden is used in the prevention and treatment of diseases [10] with the aim to support the recipients to incorporate physical activity into their everyday life [11].

Several studies have demonstrated the effectiveness of PAP to achieve increased physical activity levels in adults at 6 [5,12], 12 [12,13], 16 [8] and 24 months’ follow-up [14]. However, public health gains are affected by the uptake of PAP in routine care. Study findings suggest that PAP concepts tend to reach relatively few patients, more female recipients than male and primarily patients over the age of 45 [6,15]. In a Swedish county council-based study in 2004–2005, Leijon et al. [6] found that PAP was delivered to less than 1.5% of all primary care patients in one year. In 2007–2010, the amount of PAP in Sweden doubled, although there was a large variation in prescriptions between county councils [16]. Harrison et al. [15] estimated that 4% of the sedentary adult residents in a primary care district in northwestern England received an exercise referral in 2000–2004.

Barriers for delivery of PAP in routine care include perceived time constraints [17,18,19], reservations about the effectiveness of PAP as a treatment or preventive intervention and the lack of clear routines concerning how to integrate PAP into regular practice [18]. A Swedish study found that simplifying routines of PAP delivery for primary care physicians prescription rates increased over two years (2006–2007) [20]. Multi-professional PAP concepts that involve physical activity counsellors who provide personalized support to PAP recipients to achieve increased physical activity have been proposed as a possible means to facilitate health care delivery of PAP [18,21]. Furthermore, the use of physical activity counsellors in primary care has been proposed as a possible means to raise the quality of physical activity counselling [21,22]. However, long-term evaluations of the reach and effectiveness of multi-professional PAP concepts in routine care are lacking and there are no studies covering both primary and secondary health care settings.

Addressing important knowledge gaps with regard to PAP delivery in routine health care, this study provides a registry-based long-term evaluation of a multi-professional PAP concept with counsellor support implemented in a Swedish county council. The aim was to investigate differences in characteristics between PAP recipients and the total health care population and between PAP recipients who used counsellor support and PAP recipients who did not use this support. The aim was also to investigate health care professionals who prescribe PAP in terms of professional belonging and health care setting. The use of medical record registry data allowed for analyses of previous morbidity in all PAP recipients, which has not been done in earlier routine health care studies of PAP delivery. Furthermore, data allowed for comparisons of characteristics between PAP recipients and the total health care population.

## 2. Methods

### 2.1. Study Design and Setting

This registry-based study involved patients who received PAP in routine primary and secondary care in the County Council of Kronoberg, a predominantly rural district in southern Sweden. Health care in Sweden is primarily publicly funded. All residents are insured by the state and have equal access to health care. Out-of-pocket fees are low and regulated by law.

Primary care in the County Council of Kronoberg consists of 22 public and 11 privately operated (publicly funded) units; secondary care is provided in two public hospitals. All primary and secondary care units have used the same electronic medical record system (Cambio Cosmic, Cambio Healthcare System AB, Linköping, Sweden) since 2005.

Since 2009, patients who receive PAP in the County Council of Kronoberg are offered counselling support by appointed physical activity counsellors. Patients who want this support can contact a counsellor; there is no formal referral system. The support is free of charge and can be utilized for one year after the PAP is issued. The counsellors are health care professionals, e.g., nurses and physiotherapists, who are trained in motivational interviewing counselling techniques [23]. An important rationale for implementing the concept was to reduce the clinical work load as a means of enabling higher numbers of prescriptions of physical activity.

### 2.2. Data Collection

All patients aged 18 years or older receiving PAP in primary or secondary care in the County Council of Kronoberg in 2009–2012 were identified via the electronic medical record system using specific registry codes. The only exception was PAP delivered in two privately operated primary care centres, which were not included because of lack of consent from the managers of the centres.

In total, 5864 prescriptions were registered. Of these, 985 prescriptions were excluded because they were repeated, duplicated or erroneously registered prescriptions, such as prescriptions in home care of the elderly. Only the first prescription of each PAP recipient during the study period was selected. This left 4879 PAP recipients to be included for analysis.

The characteristics of the PAP recipients were retrieved from the electronic medical record: sex, age and all registered diagnoses for the 12 months before PAP, the number of office visits (including visits to all professions in primary and secondary care), the number of secondary inpatient care occasions, PAP-prescribing profession and prescribing health care unit.

Several measures were used to capture morbidity: registered diagnoses, office visits (to primary and/or secondary care) and inpatient (secondary) care. Diagnostic groups have previously been used to describe morbidity among frequent health care attendees [24]. The European General Practice Research Network states that any combination of at least two diseases (acute or chronic) can be used as a definition of multi-morbidity [25].

Diagnoses were grouped according to the International Classification of Diseases (ICD) system version 10 [26]. Patients were categorized according to (a) having any diagnosis versus not having any diagnosis within each diagnostic group of diseases (yes/no), and (b) the number of diagnostic groups of diseases (0–2, 3–4, ≥5). The diagnostic groups pregnancy, childbirth/puerperium and factors influencing health status and contact with health services were excluded.

The total health care population in the County Council of Kronoberg in 2009 was used as a reference population for the PAP recipients in 2009. For the reference group, data on sex, age, diagnoses and inpatient care were retrieved using the same registry codes for capturing registry data as for the PAP recipients.

### 2.3. Statistical Analysis

Data are presented using numbers and proportions (%), means and standard deviation (SD), and median and 25th and 75th percentiles. Differences between groups were tested with the chi-squared test. A *p*-value ≤ 0.05 was regarded statistically significant. All statistical analyses regarding PAP recipients were performed with IBM SPSS Statistics for Windows, version 23.0 (IBM Corp., Armonk, NY, USA). 

### 2.4. Ethical Considerations

The Regional Ethical Review Board in Linköping approved the project (Ref. No. 2013/51-31). The data were delivered in such a way that the patients and prescribers of PAP could not be identified by the researchers.

## 3. Results

### 3.1. Characteristics of PAP Recipients over Time

The 4879 adult patients who received a first PAP in primary or secondary care during 2009–2012 represents 3% of all primary and secondary care adult patients (186,117 patients) during these four years.

Approximately 60% of prescribed patients were female. The age group 45–64 years dominated (Table 1). The proportion of PAP recipients aged 65 years or older increased somewhat over time (from 26% to 33%). The mean age of the PAP recipients for the study period was 53.6 years (SD 16.1 years).

The most frequent diagnostic groups were musculoskeletal disease, circulatory disease, endocrine disease and mental health disorders (Table 1). The proportion with endocrine disease decreased from 52% in 2009 to 34% in 2012, whereas the proportion with mental health disorders increased from 23% in 2009 to 34% in 2012. The number of diagnostic groups per patient ranged from 0 to 16, with an average of 3.7 (SD 2.6). The proportion of patients with five or more diagnostic groups increased over the period, from 28% in 2009 to 34% in 2012.

During the year before PAP, more than half of the PAP recipients had 11 or more registered office visits to primary and/or secondary care and approximately 25% had been hospitalized (inpatient care) (Table 1).

Compared with the total health care population of the County Council of Kronoberg, a larger proportion of the PAP recipients were female and over 45 years old. The PAP recipients had almost twice the proportion of registered diagnoses for the majority of diagnostic groups. More PAP recipients had at least one occasion with inpatient (hospital) care, 27% compared with 14% for the total health care population (Table 2).

### 3.2. Characteristics of PAP Recipients Using Counsellor Support

One-third of all PAP recipients (*n* = 1555; 32%) used support from a physical activity counsellor in the year after prescription (Table 3). PAP recipients using support compared with non-users were more often female and over 45 years of age. The support users more often had an endocrine diagnosis and a mental health disorder, and had higher frequency of multiple diagnoses (≥5 diagnostic groups of diseases) and office visits (≥11) compared with non-users. Prescriptions by physicians were more common among counsellor support users, but no difference according to health care setting was seen.

### 3.3. PAP Delivery by Health Care Professional and Setting over Time

The total number of prescriptions increased by 20% from 2009 to 2010, but, in the fourth year, 2012, the number of prescribed patients was the same as in 2009 (Table 4). Physicians prescribed the largest proportion of PAPs, but with a decreasing proportion over the years (from 49% to 39%). In contrast, the proportion of nurses prescribing PAP increased from 22% to 30%. Physiotherapists prescribed about one-fourth of the PAP recipients in all four years. The individual variation of prescriptions was large, ranging from 1 to 135 prescriptions per prescriber (median 19, —25th to 75th percentiles (9–37). Primary care was the dominant setting with 64% to 75% of the prescriptions.

## 4. Discussion

This registry-based study investigated the characteristics of patients who received PAP and the health care professionals who issued these prescriptions in a multi-professional PAP concept with counselling support. The concept was implemented in primary and secondary care in a county council in Sweden and was studied over four years. We found higher prevalence of morbidity in terms of more diagnoses and more inpatient care among PAP recipients compared with a reference population of all patients visiting health care. Morbidity was even higher among PAP recipients who used support by PAP counsellors compared to non-users. Slightly more than half of prescriptions were by professionals other than physicians and about one-quarter were prescribed in secondary care.

All information in this registry-based study was based on data captured in electronic medical records from primary and secondary care. The very high coverage of data (almost 100%) and the length of the study period are strengths of the study. The four-year study period ensured that the results were not only an effect of enthusiasm about a new organisational structure for PAP. However, medical records are structured for use in clinical care, which means that there might be quality problems when using these data for research purposes [27]. To ensure completeness, validity, consistency and accuracy of the data, the researchers had an ongoing dialogue with health care professionals familiar with registration of codes, data analysts familiar with how to capture the specific data codes and with quality control of the county councils’ health care data, and with statisticians with experience of using health care data.

External validity, i.e., generalizability, of the study findings to other settings is somewhat restricted. Aside from sex and age, we have limited information about the PAP recipients that could facilitate comparisons between PAP populations, e.g., socio-economic variables, reason for receiving PAP, PAP recipients’ level of physical activity and motivation. Generalizability may also be restricted due to different strategies used by health care organisations to support uptake of PAP in routine care, e.g., strategies involving pay for performance of PAP, and different organisational structures for PAP delivery. On the other hand, external validity is enhanced by the fact that the study was conducted in routine care. Unlike most routine care PAP studies, the study allowed for analyses of long-term real-life health care delivery of PAP in an unselected total health care population with no selection bias. Data were collected in a way that did not require effort or even awareness of the study by the health care professionals and PAP recipients.

Furthermore, it is difficult to determine the extent to which the patients in our study differ from PAP recipients in other PAP studies with regard to morbidity because previous studies have not investigated prevalence of morbidity in terms of medical diagnoses among PAP recipients in health care populations. The high prevalence of morbidity among the PAP recipients in this study suggests that the PAP concept was predominantly applied as a secondary or tertiary preventive strategy, i.e., aimed at reducing the impact of a diagnosed disease or softening the impact of an ongoing illness [28]. The benefit of physical activity has been demonstrated in both primary (i.e., no evidence of disease) and secondary prevention [10]. However, our findings indicate that PAP is not viewed as a primary prevention strategy by the health care professionals, who instead predominantly prescribed physical activity to patients with a broad range of diseases.

The uptake of PAP in the total health care population was found to be broadly similar to what has been reported from previous population-based primary health care studies [6,15]. One-third of the PAP recipients used counsellor support. The reasons why the PAP recipients sought or did not seek counsellor support were not investigated. It is difficult to determine whether the use by one-third was a small or large proportion since there are no comparative studies. Still, the proportion of users of counsellor support was lower than expected among the health care practitioners who were involved in implementing the concept. The patients’ reasons for choosing to use, or not to use, this support will be investigated in a forthcoming study. Our findings of an association between morbidity and use of counsellor support are in line with results from a recently published study of PAP recipients with chronic musculoskeletal pain, which found that they experienced obstacles to increasing their physical activity and needed individually tailored information and support when prescribed physical activity [21].

Two-thirds of the prescriptions were issued by physicians and nurses, which is in line with a previous Swedish primary care study of PAP [6]. Persson et al. [20] found that simplified routines increased the physicians’ prescription rate over two years. In our study, in line with Leijon et al. [6], we found decreasing proportion of PAP by physicians over time. This finding suggests that the physicians’ interest or enthusiasm for the PAP concept declined over time. Physicians in Sweden have expressed some scepticism about the practice of issuing PAP [18]. Other studies have noted that physicians feel confident in providing advice about physical activity [29], but a Canadian study [7] observed that physicians were more likely to provide verbal counselling on physical activity than use PAP.

Despite the expressed ambition to achieve higher PAP rates by means of the multi-professional concept with counselling support, the prescription rates for 2012 were similar to those for 2009. However, the rates could have been even lower without the concept. Our study did not investigate the reasons for decreasing rates of prescription over time, but implementation research has shown that adoption or uptake of new practices in routine health care is influenced by a combination of several interdependent factors. These factors include the characteristics of the practices (e.g., perceived complexity and compatibility with existing routines), the health care professionals (e.g., their attitudes, beliefs, motivation and self-efficacy concerning the new practice), strategies used to facilitate the implementation and the context of the implementation [30]. Numerous factors associated with the wider context may have influenced our results. A trend towards increasing the amount of PAP in Sweden has been seen at the national level [16]. The studied PAP concept was new and it is possible that the strategies used to enhance implementation in 2009–2010, e.g., information and education targeting health care professionals, contributed to increased prescription in the initial years. However, no comparative data at the national level exist for 2011 and 2012. In 2011, the Swedish National Board of Health and Welfare introduced guidelines for disease prevention, which included recommendations for management of insufficient levels of physical activity. The effects of these guidelines on prescription rates have not been studied.

While the aim of the counsellor support was to reduce the clinical workload as a means of enabling higher numbers of prescriptions of physical activity, overall PAP rates did not increase as intended. However, it is not possible to determine how the rates would have developed without the implementation of the concept. Qualitative studies are warranted to explore some of the unanswered “why” questions of quantitative PAP research.

## 5. Conclusions

In this four-year registry-based study of a multi-professional PAP concept with counsellor support implemented in a Swedish county council, we found that PAP recipients had high morbidity and were frequent attenders in health care. Counsellor support was used by approximately one-third of all PAP recipients, and morbidity was even higher in this group. The PAP concept therefore seems predominantly to have been used as a secondary or tertiary prevention strategy. The overall prescription rate was similar to prescription rates found in other PAP studies.

## Figures and Tables

**Table 1:** Patients receiving PAP (Physical Activity on Prescription) in primary and secondary care and differences between years of prescription.

Patient Characteristics	Year				*p*-Value *
	2009	2010	2011	2012	
	*n* = 1148	*n* = 1341	*n* = 1242	*n* = 1148	
	% (*n*)	% (*n*)	% (*n*)	% (*n*)	
Sex					0.100
Female	58 (668)	63 (843)	60 (746)	62 (707)	
Male	42 (480)	37 (498)	40 (496)	38 (441)	
Age					≤0.001
18–29 years	9 (101)	9 (126)	11 (132)	11 (122)	
30–44 years	18 (209)	22 (296)	18 (221)	18 (207)	
45–64 years	48 (546)	43 (571)	43 (528)	39 (446)	
65+ years	25 (292)	26 (348)	29 (361)	33 (373)	
Diagnoses ** and health care consumption *** in the 12 months before PAP					
Musculoskeletal diseases (yes)	47 (541)	45 (602)	46 (573)	50 (574)	0.089
Endocrine diseases (yes)	52 (549)	41 (550)	38 (469)	37 (421)	≤0.001
Circulatory diseases (yes)	45 (510)	40 (529)	43 (538)	43 (497)	0.088
Mental health disorders (yes)	23 (265)	31 (412)	30 (370)	34 (391)	≤0.001
Respiratory diseases (yes)	24 (272)	26 (344)	24 (303)	27 (307)	0.298
Other diagnostic groups (yes)	76 (866)	77 (1027)	81 (999)	81 (921)	0.004
Number of diagnostic groups					0.034
0–2 diagnostic groups	35 (405)	34 (447)	33 (414)	31 (356)	
3–4 diagnostic groups	37 (425)	37 (493)	36 (442)	35 (396)	
≥5 diagnostic groups	28 (315)	29 (387)	29 (387)	34 (390)	
Number of office visits					0.054
0–5 office visits	22 (253)	23 (312)	25 (307)	27 (310)	
6–10 office visits	22 (250)	21 (282)	20 (247)	22 (255)	
≥11 office visits	56 (645)	56 (747)	55 (688)	51 (583)	
≥1 occasion of inpatient care (yes)	27 (310)	28 (376)	28 (352)	21 (245)	≤0.001

* Tested by chi-squared test. ** 24 ICD- (International Classification of Diseases) 10 diagnostic groups of diseases assessed by the first coding character, i.e., letter, excluding O (pregnancy, childbirth and the puerperium) and Z (factors influencing health status and contact with health services). Analysis of patients with (yes) or without (no) assessed diagnostic group of diseases. *** Office visits in primary and secondary care, and inpatient somatic and/or psychiatric care. Inpatient care analysis includes patients with (yes) or without (no) inpatient care.

**Table 2:** Differences in characteristics between PAP recipients and the total health care population in 2009.

Patient Characteristics	PAP Recipients	Total Health Care Population	*p*-Value *
	*n* = 1148	*n* = 121,869	
	% (*n*)	% (*n*)	
Sex			0.027
Female	58 (668)	55 (66926)	
Male	42 (480)	45 (54943)	
Age			≤0.001
18–29 years	9 (101)	18 (22515)	
30–44 years	18 (209)	29 (35392)	
45–64 years	48 (546)	24 (29752)	
65+ years	25 (292)	28 (34210)	
Diagnostic groups **			
Musculoskeletal diseases (yes)	47 (541)	25 (30753)	≤0.001
Endocrine diseases (yes)	48 (549)	13 (15443)	≤0.001
Circulatory diseases (yes)	45 (510)	21 (26089)	≤0.001
Mental health disorder (yes)	23 (265)	11 (13464)	≤0.001
Respiratory diseases (yes)	24 (272)	18 (22372)	≤0.001
Other diagnostic groups (yes)	76 (866)	65 (79366)	≤0.001
Inpatient care ***			≤0.001
≥1 occasion of inpatient care	27 (310)	14 (2399)	

* Tested by chi-squared test. ** 24 ICD-10 diagnostic groups of diseases assessed by the first coding character, i.e., letter, excluding O (pregnancy, childbirth and the puerperium) and Z (factors influencing health status and contact with health services). Analysis of patients with (yes) or without (no) assessed diagnostic group of diseases. PAP recipients’ diagnoses were measured in the 12 months before PAP. *** Somatic and/or psychiatric inpatient care within a year before PAP. PAP recipients’ inpatient care was measured in the 12 months before PAP. Analysis includes patients with (yes) or without (no) inpatient care.

**Table 3:** Differences in characteristics between patients using versus not using counsellor support after receiving a PAP (*n* = 4879).

Patient Characteristics	Counsellor Support		*p*-Value *
	Yes	No	
	(*n* = 1555)	(*n* = 3324)	
	% (*n*)	% (*n*)	
Sex			≤0.001
Female	66 (1024)	58 (1940)	
Male	34 (531)	42 (1384)	
Age			≤0.001
18–29 years	8 (125)	11 (356)	
30–44 years	20 (316)	19 (617)	
45–64 years	46 (712)	41(1379)	
65+ years	26 (402)	29 (972)	
Diagnoses ** and health care consumption *** in the 12 months before PAP			
Musculoskeletal diseases (yes)	49 (749)	47 (1541)	0.221
Endocrine diseases (yes)	44 (683)	39 (1306)	0.002
Circulatory diseases (yes)	42 (645)	43 (1429)	0.337
Mental health disorder (yes)	31 (487)	29 (951)	0.050
Respiratory diseases (yes)	27 (409)	25 (817)	0.187
Other diagnostic groups (yes)	79 (1218)	79 (2595)	0.773
Number of diagnostic groups			0.011
0–2 diagnostic groups	29 (450)	34 (1102)	
3–4 diagnostic groups	37 (566)	36 (1190)	
≥5 diagnostic groups	33 (509)	30 (965)	
Number of office visits			0.002
1–5 office visits	23 (354)	25 (828)	
6–10 office visits	19 (295)	22 (739)	
≥11 office visits	58 (906)	53 (1757)	
≥1 occasion of inpatient care	25 (389)	27 (893)	0.171
Prescribing professional			≤0.001
Physician	49 (767)	41(1356)	
Nurse	23 (357)	28 (935)	
Physiotherapist	19 (295)	24 (799)	
Other professionals ****	9 (136)	7 (234)	
Prescribing setting			0.082
Primary care	70 (1093)	70 (2345)	
Secondary somatic care	25 (392)	24 (783)	
Secondary psychiatric care	5 (70)	6 (196)	

* Tested by chi-squared test. ** 24 ICD-10 diagnostic groups of diseases assessed by the first coding character, i.e., letter, excluding O (pregnancy, childbirth and the puerperium) and Z (factors influencing health status and contact with health services). Analysis of patients with (yes) or without (no) assessed diagnostic group of diseases. *** Office visits in primary and secondary care, and inpatient somatic and/or psychiatric care. Inpatient care analysis includes patients with (yes) or without (no) inpatient care. **** Psychologists, behavioural therapists, midwifes, dieticians and occupational therapists.

**Table 4:** PAP delivery by health care professional and setting over time (*n* = 4879).

Professional Groups and Health Care Setting	Year of Prescription				*p*-Value *
	2009	2010	2011	2012	
	*n* = 1148	*n* = 1341	*n* = 1242	*n* = 1148	
	% (*n*)	% (*n*)	% (*n*)	% (*n*)	
Professional group					≤0.001
Physician	49 (566)	47 (626)	39 (483)	39 (448)	
Nurse	22 (253)	24 (322)	30 (374)	30 (343)	
Physiotherapist	22 (254)	21 (280)	23 (286)	24 (274)	
Other professionals **	7 (75)	8 (113)	8 (99)	7 (83)	
Health care setting					≤0.001
Primary care	74 (850)	70 (941)	64 (791)	75 (856)	
Secondary somatic care	24 (277)	23 (308)	31 (391)	17 (199)	
Secondary psychiatric care	2 (21)	7(92)	5 (60)	8 (93)	

* Tested by chi-squared test. ** Psychologists, behavioural therapists, midwifes, dieticians, occupational therapists.
